# Antibacterial and Anti-Inflammatory Activities of *Moringa oleifera*: Extracts, Bioactive Compounds, and Mechanisms

**DOI:** 10.3390/ijms27198633

**Published:** 2026-09-26

**Authors:** Mingxia Xiao, Leng Wang, Zubing Zhang, Haiquan Li, Baocai Gao

**Affiliations:** Yunnan Technology Innovation Center of Natural Rubber, Yunnan Key Laboratory of Sustainable Utilization Research on Rubber Tree, Yunnan Institute of Tropical Crops, Jinghong 666100, China; mmswxxa8346@163.com (M.X.);

**Keywords:** *Moringa oleifera*, antibacterial activity, anti-inflammatory activity, isothiocyanate, NF-κB, Nrf2, antimicrobial resistance

## Abstract

*Moringa oleifera* (Moringaceae) is a tropical tree whose leaves and stems have long been used in South Asian and African traditional medicine for infectious and inflammatory disorders. These tissues contain a diverse array of secondary metabolites, chiefly flavonoids, phenolic acids, isothiocyanates, sterols, and peptides, and exhibit broad-spectrum antibacterial activity against Gram-positive and Gram-negative bacteria, including multidrug-resistant strains. Their anti-inflammatory effects have been attributed to the suppression of nuclear factor-κB (NF-κB)/Toll-like receptor 4 (TLR4) signaling and activation of the NF-E2-related factor 2 (Nrf2)/Kelch-like ECH-associated protein 1 (Keap1) antioxidant axis. This review synthesizes the phytochemistry of *Moringa* leaves and stems and critically evaluates current evidence for their antibacterial and anti-inflammatory activities, molecular targets, and potential crosstalk between the two. We further discuss extraction methods, structure–activity relationships, pharmacokinetics, safety, and applications spanning medicine, animal production, food preservation, and cosmetics. The evidence base is heterogeneous owing to variation in plant origin, extraction protocols, bacterial strains, and assay design; mechanistic and dosing data specific to leaves and stems remain preliminary. Across reports, leaf and stem extracts inhibit common Gram-positive and Gram-negative pathogens at MICs mostly in the approximate 0.2–4 mg/mL range, and isolated flavonoids and glucosinolate isothiocyanates act on bacterial membranes and quorum sensing and on host NF-κB/Nrf2 signaling; however, no study has formally quantified antibacterial–anti-inflammatory synergy within one assay system. Key gaps include standardized preparations, direct tests of antibacterial–anti-inflammatory synergy, systematic pharmacokinetic/pharmacodynamic and long-term safety studies, and high-quality clinical trials.

## 1. Introduction

### 1.1. Botanical Characteristics and Traditional Medicinal Use

*Moringa oleifera* is a perennial, tropical, deciduous tree in the monogeneric family Moringaceae, which contains the single genus *Moringa*. Some 13 *Moringa* species are recognized, most of them found in the semiarid tropics that stretch from Africa to Asia. *M. oleifera* is native to the northern Indian subcontinent, on the southern slopes of the Himalayas, and is the most widely cultivated and studied species because of its adaptability, high biomass, and versatility [1,2]. It is a fast-growing tree reaching 3–12 m in height, with soft, corky bark and branches bearing conspicuous lenticels and leaf scars. Its tripinnate leaves are 25–60 cm long and bear thin, ovate to elliptic leaflets; spreading panicles bear fragrant, yellowish-white flowers; and the slender, three-angled capsule, 20–50 cm long, resembles a drumstick, from which the tree takes its common name [2,3]. A warm-adapted, light-demanding species that tolerates drought and grows well at 25–35 °C, *M. oleifera* is now cultivated throughout tropical and subtropical regions, including India, Africa, and Southeast Asia, and in the Chinese provinces of Yunnan, Hainan, Guangdong, Guangxi, Fujian, and Taiwan [1,2].

Nearly every part of the plant is used: the leaves, flowers, tender pods, seeds, bark, and roots all hold food or medicinal value. In Ayurveda, *M. oleifera* (Sanskrit *Shigru*) is described as pungent and bitter, with a hot potency (*Ushna virya*) that balances *Kapha* and *Vata*. It is credited with anti-inflammatory and decongestant (*Shothahara*), anthelmintic (*Krimighna*), and appetizing and digestive (*Deepana*) actions, and has been used for edema, rheumatoid arthritis, digestive disorders, parasitic infections, and respiratory disease [1,4]. In the traditional medicine of Nigeria and other parts of Africa, the leaves are used for stomach pain, diarrhea, malaria, and malnutrition; bark decoctions, for fever, toothache, and urinary infection; and seed oil, applied externally, for rheumatic pain and skin infection [5].

### 1.2. Background

Antimicrobial resistance has become a major challenge to global public health. The World Health Organization reports that the continuing spread of MDR bacteria erodes the efficacy of commonly used antibiotics, driving interest in naturally derived, low-toxicity antibacterial agents [6]. Chronic low-grade inflammation is likewise recognized as a common pathological basis for cardiovascular disease, diabetes, chronic kidney disease, arthritis, and cancer. Long-term use of nonsteroidal anti-inflammatory drugs (NSAIDs) and glucocorticoids is associated with adverse effects, including gastrointestinal injury and hepatic and renal dysfunction, leaving an unmet clinical need for safe and effective natural anti-inflammatory agents [7,8].

In this setting, the broad-spectrum antibacterial and anti-inflammatory activities of *M. oleifera* leaves and stems have drawn growing interest. Leaf and stem extracts do more than inhibit or kill bacteria: several studies report that they also suppress virulence-factor secretion and biofilm formation, effects that are proposed to lower the selection pressure [9]. Their anti-inflammatory activity has been attributed to the multitarget regulation of host immune responses and is proposed to act in concert with their antibacterial action to confer coordinated protection [10]. A structured synthesis of the evidence for these two activities in leaves and stems is therefore needed to guide their development as medicines (Figure 1).

### 1.3. Research Gaps, Scope, and Contributions of This Review

The pharmacological literature on *M. oleifera* has expanded rapidly but unevenly. The literature on anti-inflammatory mechanisms is comparatively well-developed from in vitro and animal mechanistic studies. Chis et al. [4] describe how quercetin, kaempferol, and related compounds inhibit cyclooxygenase (COX) and lipoxygenase (LOX), block NF-κB nuclear translocation, and downregulate pro-inflammatory cytokines such as tumor necrosis factor-α (TNF-α) and interleukin-1β (IL-1β). Mohd Sahardi and Makpol [11], writing from an inflammaging perspective, review evidence that *M. oleifera* inhibits NF-κB signaling, lowers TNF-α and IL-6, and enhances antioxidant–enzyme activity. Dedicated antibacterial reviews remain scarce, and the relevant material is scattered across broad surveys [2,3,12] that tend to report inhibition-zone and minimum inhibitory concentration (MIC) values strain by strain, without integrating resistance mechanisms or compound–target correspondence.

Relative to these earlier syntheses, our contribution is threefold. First, we examine the interplay between antibacterial and anti-inflammatory activities, how they may act sequentially during infection and how individual constituents may reinforce one another, distinguishing demonstrated interactions from mechanistic hypotheses. Second, we grade mechanistic evidence hierarchically, from molecular-docking predictions through in vitro enzyme and cell assays to animal and human studies, enabling readers to gauge the strength of each conclusion. Third, we analyze how extraction method, plant origin, and strain differences drive heterogeneity in key parameters such as MIC, and we critically assess limitations and future directions. Accordingly, this Review covers the chemical constituents of leaves and stems; both activities and their mechanisms; isolation and structure–activity relationships; and in vivo metabolism and safety, with the goal of providing an evidence-based reference and research roadmap for medicinal development, standardized preparations, and clinical translation.

### 1.4. Literature Search Strategy and Selection Criteria

The literature was retrieved from PubMed/MEDLINE, Web of Science, Scopus, and Google Scholar up to August 2026, using combinations of the search terms “*Moringa oleifera*” with “antibacterial”, “antimicrobial”, “anti-inflammatory”, “isothiocyanate”, “flavonoid”, “mechanism”, “biofilm”, “pharmacokinetic”, and “toxicity”. Peer-reviewed original research and reviews in English were considered; after duplicate removal and title/abstract screening for relevance to leaves and stems, 134 references were retained. Evidence obtained primarily from seeds is explicitly labeled “seed-derived”. Because this is a narrative rather than a systematic review, no quantitative risk-of-bias appraisal was performed. The selection flow is summarized in Figure 2.

## 2. Chemical Constituents of Leaves and Stems

The pharmacological activity of *M. oleifera* leaves and stems arises from a rich complement of secondary metabolites. and the constituents more than 160 compounds have been reported across *M. oleifera* as a whole plant (all tissues combined), although only a smaller subset has documented bioactivity; the constituents most relevant to the two activities in leaves and stems fall into five major classes [13,14] (Table 1). Throughout this Review, evidence obtained from seeds rather than from leaves or stems is explicitly labeled “seed-derived”: several signature compounds, most notably the ITCs and the chitin-binding peptides, were first characterized in seeds and have yet to be quantified systematically in leaves and stems.

### 2.1. Flavonoids

Flavonoids are the most abundant and best-characterized active constituents of leaves and stems. More than 30 flavonoids have been isolated, chiefly quercetin, kaempferol, astragalin, isoquercitrin, rutin, and myricetin, together with their malonylated glycoside derivatives [5,13]. Leaves reportedly contain a wider variety and higher amounts of flavonoids than stems, whereas stems are dominated by the more polar flavonoid glycosides, which extract more readily in water or aqueous alcohol [13]. Their proposed antibacterial actions include the disruption of membrane integrity, inhibition of bacterial enzymes, and interference with the electron-transport chain; their anti-inflammatory actions include the inhibition of NF-κB signaling, reduced secretion of pro-inflammatory cytokines, direct radical scavenging, and transition-metal chelation [4].

### 2.2. Phenolic Acids

The phenolic acids of leaves and stems include chlorogenic, caffeic, gallic, p-coumaric, and ferulic acids; chlorogenic, caffeic, and gallic acids occur at relatively high levels and are the best characterized [13]. Phenolic acids can perturb bacterial membrane integrity and thereby facilitate the entry of flavonoids. On the host side, the two classes act at several nodes of the NF-κB pathway. Such class-level cooperation is biochemically plausible, but direct, quantitative evidence for phenolic-acid/flavonoid synergy in *M. oleifera* remains limited [16].

### 2.3. Isothiocyanates and Glucosinolates

ITCs are the most distinctive sulfur-containing constituents. They do not exist as such in intact tissue but are released when glucosinolates are hydrolyzed by myrosinase or by the gut microbiota. The most abundant glucosinolate is glucomoringin (GMG), whose hydrolysis product is glucomoringin isothiocyanate (GMG-ITC), also termed moringin and abbreviated MIC-1 in some earlier papers (chemical name: 4-(α-L-rhamnopyranosyloxy) benzyl isothiocyanate) [17,18]. To avoid confusion with the minimum inhibitory concentration (MIC), this Review uses “GMG-ITC (moringin)” throughout and does not use the MIC-1 abbreviation. It is one of the most extensively investigated anti-inflammatory monomers in *Moringa oleifera*. This compound has been reported to activate the cytoprotective Nrf2/Keap1 pathway, induce NQO1, HO-1, and GSTP1, and inhibit NF-κB nuclear translocation. In several seed-derived preparations, its activity was benchmarked against curcumin as a comparator (seed-derived evidence) [17,19,20]. As antibacterial agents, ITCs have been reported to inhibit biofilm formation, perturb membrane integrity, and inhibit resistance-related enzymes [9]. Glucomoringin and moringin, together with the Mo-CBP chitin-binding proteins described in Section 2.5, are regarded as chemotaxonomic marker constituents that were first isolated from and are most concentrated and best characterized in *M. oleifera*; they are not strictly exclusive to the species and also occur, usually at lower levels, in related Moringa species.

### 2.4. Terpenoids and Sterols

The terpenoids and sterols of leaves and stems include β-sitosterol, stigmasterol, and lupeol. These relatively nonpolar compounds are enriched more efficiently in lipid-soluble solvents such as chloroform and ethyl acetate, consistent with the generally stronger antibacterial activity reported for organic versus aqueous extracts [21,22], although the relative activity of organic and aqueous extracts is strain- and protocol-dependent and does not follow a fixed hierarchy [22]. Wizrah et al. [23] compared two Moringa species from the Wadi Ad-Dawasir region of Saudi Arabia. By GC–MS, an ethanol extract of *M. peregrina* leaves was rich in stigmasterol and β-sitosterol, whereas an *M. oleifera* leaf ethanol extract was dominated by fatty acids, cis-vaccenic stearic, and linoleic acids, and contained substantially lower levels of these phytosterols. Both ethanol extracts inhibited all four Gram-negative strains tested (*E. coli*, *P. aeruginosa*, *Sh. sonnei*, and *Sh. shiga*) at an MIC of 50 μg/mL, the lowest concentration assayed [23].

### 2.5. Peptides and Other Constituents

*M. oleifera* contains distinctive chitin-binding proteins, including Mo-CBP2, Mo-CBP3, and Mo-CBP4. These proteins have so far been isolated and purified mainly from seeds, and their abundance and activity in leaves and stems remain to be characterized systematically (seed-derived) [24,25]. Mo-CBP2 showed antifungal activity against *Candida albicans* by increasing membrane permeability and inducing reactive oxygen species (ROS) [24]. Synthetic peptides designed from Mo-CBP3 (Mo-CBP3-PepI/II/III) were active against several human pathogenic bacteria, stimulating ROS generation and increasing plasma-membrane permeability [25]. Leaves and stems also contain fatty acids (oleic, linoleic, and palmitic), alkaloids, and vitamins that may contribute additively to antioxidant and immunomodulatory effects [14,26].

## 3. Antibacterial Activity

### 3.1. Broad-Spectrum Activity of Extracts

In vitro studies report that leaf and stem extracts inhibit both Gram-positive bacteria (*Staphylococcus aureus*, *Bacillus cereus*, and *Enterococcus faecalis*) and Gram-negative bacteria (*E. coli*, *P. aeruginosa*, *Klebsiella pneumoniae*, and *Salmonella enterica* serovar Typhi), with some activity against fungi as well [14,24]. The extraction solvent has a marked effect: the extraction solvent strongly affects activity because organic and aqueous solvents enrich different constituents (lipid-soluble aglycones, ITCs, and sterols versus polar glycosides and phenolic acids); the direction of the difference is strain-dependent and aqueous preparations are occasionally more potent [22,23]. Most quantitative MIC data come from leaves rather than stems, and no study has compared leaf and stem extracts under identical extraction and assay conditions, so a definitive leaf-versus-stem activity ranking cannot yet be made [16,21]. Leaf extracts, together with seed extracts (the latter explicitly seed-derived), inhibit antibiotic-resistant isolates, including methicillin-resistant S. aureus (MRSA) and MDR Gram-negative strains; the supporting study examined leaf and seed rather than leaf and stem material [27].

### 3.2. Activity of Isolated Compounds

Several antibacterial monomers have been isolated from, or tested in, *M. oleifera* material (Table 2). In a head-to-head comparison of four *M. oleifera* polyphenol monomers against *E. coli*, astragalin had an IC50 of 490 μmol/L (approximately 0.22 mg/mL), followed in that comparison by rutin (588 μmol/L, approximately 0.36 mg/mL), chlorogenic acid (605 μmol/L, approximately 0.21 mg/mL), and hyperoside (676 μmol/L, approximately 0.31 mg/mL); astragalin also inhibited biofilm formation and initial adhesion and compromised membrane integrity [15]. Quercetin and kaempferol inhibit *S. aureus*, *E. faecalis*, *P. aeruginosa*, and *K. pneumoniae*; docking predicts binding to bacterial DNA gyrase and topoisomerase IV, which could interfere with DNA replication, although direct confirmation of enzyme inhibition by compounds isolated from *M. oleifera* leaves or stems is still lacking: these are computational predictions, and one cited study modeled flavonoid-coated gold nanoparticles rather than a free leaf constituent; a recent leaf-extract docking-and-assay study likewise provides prediction rather than direct enzyme kinetics [28,29,30]. GMG-ITC has been reported to inhibit *E. coli*, *S. aureus*, and *P. aeruginosa*, with activity tied mainly to biofilm and quorum-sensing pathways [9,17,31]. β-Sitosterol and stigmasterol are reported to insert into the bacterial phospholipid bilayer, compromise its integrity with leakage of cellular contents, and act additively with flavonoids [23].

### 3.3. Mechanisms of Antibacterial Action

Antibacterial action arises from a multitarget network that spans bacterial structure, metabolic enzymes, virulence-factor expression, and quorum-sensing regulation, and these mechanisms can act in concert. On current evidence, themes they fall into four themes:

1. Disruption of membrane and cell-wall integrity. As amphipathic molecules, sterols (β-sitosterol and stigmasterol) interact with the phospholipid bilayer and perturb its fluidity and permeability, causing leakage of intracellular potassium, proteins, and nucleic acids [23]. Scanning electron microscopy revealed membrane wrinkling, surface indentation, and leakage in *Sh. sonnei* treated with an ethanol/aqueous extract [23]. The flocculating *M. oleifera* cationic protein/polypeptide (MOCP), a seed-derived protein distinct from the chitin-binding Mo-CBP family, disrupts bacterial membranes by inducing membrane fusion (seed-derived) [34]; the native chitin-binding proteins Mo-CBP2, Mo-CBP3, and Mo-CBP4 target mainly fungal cell-wall chitin with only limited direct antibacterial support, whereas Mo-CBP3-PepI/II/III are synthetic designed peptides, not native leaf or stem proteins [24,25]. Fatty acids such as oleic and linoleic acid add surfactant-like membrane injury [14]. Membrane damage by lipid-soluble constituents may facilitate the entry of water-soluble flavonoid glycosides and phenolic acids; this offers a plausible basis for the broader activity of crude extracts relative to isolated compounds, although time-resolved, stepwise verification is still lacking.
2. Inhibition of metabolic enzymes and macromolecular synthesis. In docking studies, flavonoids (quercetin and kaempferol) are predicted to interact with the ATP-binding sites of bacterial DNA gyrase (GyrB) and topoisomerase IV (ParE), potentially blocking DNA replication and transcription; reported binding energies vary among studies and call for confirmation by enzyme assays [35]. The –N=C=S group of GMG-ITC can react with protein cysteine thiols, and on this basis GMG-ITC has been proposed to inhibit energy-metabolism enzymes such as succinate dehydrogenase and NADH dehydrogenase and to deplete ATP; direct enzymological confirmation for *M. oleifera* ITCs is still lacking [9,17,36]. Phenolic acids may interfere with cell-wall synthesis and ribosomal function, but direct evidence remains limited [16].
3. Inhibition of virulence factors and biofilms. Unlike antibiotics that target processes essential for survival, several *M. oleifera* constituents suppress virulence-factor expression and biofilm formation at subinhibitory concentrations, an antivirulence strategy proposed to lower selection pressure. ITCs inhibit quorum-sensing (QS) signaling and reduce the extracellular polymeric substances matrix, rhamnolipid production, and motility; the mechanistic QS evidence in ref. [37] concerns iberin from horseradish rather than moringin, so direct confirmation in *M. oleifera* ITCs remains incomplete [37]. Flavonoids (quercetin and kaempferol) inhibit the *S. aureus* agr system and reduce α-hemolysin and enterotoxin secretion [4]. Astragalin inhibits *E. coli* biofilm formation and initial adhesion at subinhibitory concentrations, with an IC50 of 490 μmol/L [15]. Biofilm-embedded bacteria can be markedly more tolerant than planktonic cells [38]; whether *M. oleifera* extracts inhibit the biofilms of MRSA and extended-spectrum β-lactamase (ESBL)-producing *P. aeruginosa* at subinhibitory concentrations still requires primary confirmation [23]. Such antivirulence activity, if confirmed, would be relevant to chronic, biofilm-associated MDR infections [36].
4. Multicomponent cooperation and antibiotic sensitization. Lipid-soluble sterols and fatty acids may first increase bacterial-membrane permeability, facilitating the entry of water-soluble flavonoids and phenolic acids [9,16,39]. ITCs can then inhibit biofilms and expose deeper bacterial layers. Flavonoids may further suppress efflux-pump activity, reducing the export of intracellular compounds. Leaf and stem extracts may also act additively with conventional antibiotics, but the evidence is part- and antibiotic-specific: to date, synergistic checkerboard data are available only for a seed methanolic extract combined with β-lactams (ampicillin, cephalexin, and amoxicillin–clavulanate) against MDR *E. coli* isolated from street-vended food [40]. Separately, a stem bark extract combined with ampicillin against MRSA showed checkerboard and time–kill synergy (FICI ≤ 0.237) via β-lactamase inhibition and PBP2a downregulation [41]. Whether leaf extracts, other antibiotic classes, or in vivo settings achieve comparable synergy remains untested by standardized checkerboard/time–kill assays.

### 3.4. In Vivo Animal Studies and Sources of Heterogeneity

In vivo antibacterial activity has been reported in several animal models. In a rat model of infected diabetic ulcers, an ointment prepared from a leaf methanol extract promoted healing of ulcers infected with MRSA or *P. aeruginosa*; over 14 days, scab formation and epithelialized area increased while bacterial load fell in a dose-dependent manner [32]. In systemic-infection models, a leaf ethanol extract was active against systemic mouse infections caused by *E. coli*, *S. aureus*, and *P. aeruginosa* [14]. Reported MICs can differ markedly among studies for several reasons. (1) Extraction: solvent, temperature, solid-to-liquid ratio, and extraction time alter active-compound composition, and solvent polarity changes both yield and potency, with no universally superior solvent [23]. (2) Plant origin: geography, season, plant part, and cultivar alter content and activity; for example, ethanol and aqueous extracts of *M. oleifera* and *M. peregrina* from Wadi Ad-Dawasir predominantly showed MICs of 50 μg/mL against four Gram-negative strains, the sole deviation being the *M. oleifera* cold aqueous extract against *P. aeruginosa* (100 μg/mL) [23], whereas extracts from many other regions fell in the mg/mL range [16,21,22]. (3) Strain differences: source, passage, and susceptibility profile differ, and clinical isolates are usually more resistant than reference strains. (4) Method: MIC assay (broth or agar dilution versus disk diffusion), medium, inoculum, and incubation conditions all affect the result. Standardized extraction and susceptibility protocols are therefore needed to ensure comparability and reproducibility.

## 4. Anti-Inflammatory Activity

### 4.1. Activity of Extracts

Leaf and stem extracts inhibit acute and chronic inflammation induced by lipopolysaccharide (LPS), cigarette-smoke extract, carrageenan, and other stimuli [4,42]. Published comparative anti-inflammatory bioassays cover mostly leaves, seeds, and roots, and direct side-by-side comparisons of leaf and stem extracts under identical extraction conditions (NO, TNF-α, and IL-6 in LPS-challenged macrophages) are still lacking. The reported hierarchy, with leaves most active and stalks least, was derived from antioxidant assays rather than dedicated anti-inflammatory endpoints [43,44]. The aqueous extract of *M. oleifera* leaves showed dose-dependent anti-inflammatory activity comparable to diclofenac and accelerated wound healing in multiple rodent models [45]. Consistent with the observation that low molecular weight peptides confer stronger anti-inflammatory effects, Alcalase-hydrolyzed *Moringa oleifera* leaf protein exhibited superior inhibition of NO, TNFα, and IL6 compared to intact protein in LPS-challenged RAW264.7 macrophages [46].

### 4.2. Activity of Isolated Compounds

GMG-ITC (moringin) is the most thoroughly studied anti-inflammatory monomer. It promotes Nrf2 nuclear translocation and inhibits IκB phosphorylation and NF-κB nuclear translocation, achieving dual regulation through Nrf2/Keap1 activation and NF-κB inhibition; in carrageenan paw-edema and LPS-sepsis models, its activity was benchmarked against a curcumin comparator (largely seed-derived evidence) [17,19]. Flavonoids (quercetin, kaempferol, astragalin, and isoquercitrin) act by inhibiting NF-κB, reducing pro-inflammatory cytokine release, inhibiting COX/LOX, and scavenging radicals [4]. Phenolic acids (gallic, caffeic, and chlorogenic) use related mechanisms, with generally weaker reported activity [16].

### 4.3. Mechanisms of Anti-Inflammatory Action

Anti-inflammatory action draws on a network of several pathways, cell types, and targets. Unlike NSAIDs, which mainly target COX, these constituents act at several levels: they block receptor-proximal signaling (TLR4/MyD88 and IKK activation), inhibit the core IκBα/NF-κB cascade, modulate downstream cytokine and enzyme effectors (iNOS, COX-2, and MMPs), and activate the endogenous Nrf2 antioxidant defense. On current evidence, the action resolves into four dimensions (Figure 3).

1. Inhibition of the NF-κB/TLR4 pro-inflammatory pathway. NF-κB is the central transcription factor of the inflammatory response, and its excessive activation is shared across many inflammatory diseases [47,48]. In resting cells, NF-κB is retained in the cytoplasm by IκB-α; on stimulation by LPS or TNF-α, the IκB kinase (IKK) complex phosphorylates IκB-α and targets it for degradation, freeing the p65/p50 dimer to enter the nucleus and transcribe TNF-α, IL-1β, IL-6, inducible nitric oxide synthase (iNOS), and COX-2 [4]. Several constituents intervene at multiple nodes. At the receptor-proximal level, phenolic acids have been suggested to inhibit TLR4 dimerization and MyD88 recruitment in silico and in vitro [16]. Further downstream, GMG-ITC (seed-derived) has been suggested, by extrapolation from the demonstrated covalent modification of IKKβ Cys179 by related isothiocyanates such as sulforaphane, to modify this residue and suppress IKK activity; this specific interaction has not been demonstrated directly for moringin [17,19], whereas quercetin and kaempferol inhibit the DNA binding and nuclear translocation of the p65 subunit [4]. These molecular predictions are consistent with in vivo Western-blot data showing reduced phosphorylated IκB-α and nuclear p65, together with accumulated cytoplasmic IκB-α, in inflamed tissue [19,49,50,51].
2. Activation of the Nrf2/Keap1 antioxidant pathway. Nrf2 is the master transcriptional regulator of the cellular antioxidant response [52,53,54]. In resting cells, it is bound to Keap1 and targeted for degradation; under oxidative or electrophilic stress, Keap1 cysteines are modified, Nrf2 is released and enters the nucleus, and binding to the antioxidant response element (ARE) induces NQO1, HO-1, GSTP1, SOD, and catalase [17,20]. GMG-ITC activates Nrf2: its –N=C=S group modifies key Keap1 cysteines (Cys151, Cys273, and Cys288), dissociates the Nrf2–Keap1 complex, and stabilizes Nrf2 (seed-derived) [17,19]; it also upregulates NQO1 and HO-1 while lowering intracellular ROS and mitochondrial superoxide, with the precise fold induction varying by system [19,55]. Flavonoids likewise promote Nrf2 phosphorylation and nuclear translocation through PI3K/Akt [4]. Beyond reducing oxidative injury, Nrf2 activation may indirectly suppress inflammation by limiting ROS-mediated NF-κB activation, closing a protective feedback loop.
3. Inhibition of pro-inflammatory enzymes and mediators. Beyond transcriptional control, flavonoids, with quercetin as the leading example, inhibit COX-1/COX-2 and 5-lipoxygenase (5-LOX), reducing prostaglandin E2 (PGE2) and leukotriene B4 (LTB4); COX-2 inhibition occurs at micromolar concentrations, and potency relative to synthetic NSAIDs varies among assays [4]. Rutin acts mainly by downregulating COX-2 protein expression rather than by direct competitive inhibition [4,56]. ITCs downregulate iNOS at the transcriptional and translational levels, reduce excess NO, and limit peroxynitrite (ONOO^−^) formation and nitrative damage; these effects are linked to Nrf2/Keap1 activation and NF-κB inhibition (seed-derived) [17,57]. Phenolic acids reduce the induced expression of arachidonic-acid metabolic enzymes through radical scavenging and NF-κB inhibition [4,16]. Leaf extract and an isothiocyanate nanoliposome formulation down-regulated MMP-1 in UV-irradiated human keratinocytes [58,59], whereas a leaf-derived oleamide attenuated TGF-β1/SMAD2/3 profibrotic signaling, together limiting tissue degradation and abnormal remodeling [60].
4. Bidirectional regulation of macrophage polarization and other immune cells. Macrophages polarize toward a pro-inflammatory M1 phenotype (TNF-α, IL-1β, IL-6, and iNOS) or an anti-inflammatory M2 phenotype (IL-10, TGF-β, and arginase-1) [61,62,63]. Leaf and stem extracts inhibit LPS-induced M1 polarization, lower iNOS and pro-inflammatory cytokines, and induce IL-10, a pattern consistent in those reports with a shift toward an anti-inflammatory phenotype [17,64]. The extracts also inhibit neutrophil chemotaxis and degranulation (elastase and myeloperoxidase), inhibit mast-cell degranulation (histamine and tryptase), and skew T-cell subsets toward regulatory T cells [4,50,65]. Such control across multiple cell types may restrain pathological inflammation without abolishing normal host defense.

### 4.4. In Vivo Animal Studies

In noninfectious inflammation models, leaf extracts showed activity in carrageenan rat paw edema, cigarette-smoke acute lung injury in mice, acetic-acid colitis, and gentamicin kidney injury [4]. In infectious-inflammation models, activity was reported in *S. aureus*-infected rat skin ulcers and in the systemic inflammation of *P. aeruginosa*-infected mice [32]. Western blotting showed the inhibition of NF-κB p65 nuclear translocation (with reduced phosphorylated IκBα and accumulated cytoplasmic IκBα) and upregulation of Nrf2 protein in inflamed tissue [19].

### 4.5. Human Evidence

Dedicated human evidence for the anti-inflammatory activity of *M. oleifera* leaves and stems remains limited and heterogeneous, and no large, multicenter, randomized double-blind trial for an anti-inflammatory or anti-infective indication has been reported. Existing human studies of oral *M. oleifera* leaf powder or extract have focused mainly on metabolic markers and nutrition, with small samples and variable doses; anti-inflammatory efficacy has not been demonstrated in these studies; a double-blind randomized trial in adults living with HIV examined leaf-powder supplementation, and a narrative review of human studies found short-term use generally tolerated, although doses, preparations, and follow-up were heterogeneous and no pooled safety estimate is available [66,67]. Registered trials illustrate this focus: NCT04734132 (NUTRIMOL-DB) examined leaf effects on glycemia, lipemia, and the inflammatory profile in prediabetic participants (completed); NCT05861076 assessed acceptability and side effects of leaf powder in healthy adults (completed); NCT05002881 (E-HS-01) examined flow-mediated dilatation and hemodynamics; and NCT05191069 tested a *M. oleifera* mouthwash in orthodontic patients. None was powered primarily on an anti-inflammatory or anti-infective endpoint, and the registries otherwise concentrate on metabolic and nutritional indications.

Clinical translation therefore appears to be at an early stage and will require properly registered, adequately powered trials.

## 5. Interplay Between Antibacterial and Anti-Inflammatory Actions

The combined antibacterial and anti-inflammatory profile offer a potential advantage for the adjunctive management of infectious disease and sets *M. oleifera* apart from a single antibiotic or a single anti-inflammatory drug [2,68]. During bacterial infection, pathogen proliferation and the host inflammatory response interact dynamically. Pathogen-associated molecular patterns (PAMPs), such as Gram-negative LPS and Gram-positive peptidoglycan, activate innate immunity through pattern-recognition receptors such as TLR4/TLR2, triggering NF-κB signaling and a TNF-α/IL-1β/IL-6 cascade [69]. Excessive or sustained inflammation can cause tissue injury and increase susceptibility to secondary infection, driving an infection–inflammation–injury cycle that can progress to sepsis and multiple-organ dysfunction [70]. Because leaves and stems contain flavonoids, ITCs, and phenolic acids that act at several targets, inhibiting bacterial growth, blocking NF-κB, and activating Nrf2, they could in principle intervene at several points of this cycle at once [3]. This section examines the proposed interplay at four levels (constituents, pathways, time, and therapeutics) and distinguishes demonstrated interaction from hypothesis (Figure 4).

### 5.1. Antibacterial Action Controls the Source of Infection

Active constituents inhibit bacteria through the cooperating mechanisms described in Section 3, removing the central inflammatory stimulus. The antivirulence strategy is distinctive: at subinhibitory concentrations, ITCs and flavonoids may suppress QS and virulence-factor secretion, lowering pathogenicity without strong bactericidal pressure and thereby limiting resistance selection [9]. Activity against resistant isolates and possible antibiotic sensitization add value in resistant infection, but the supporting data are part- and antibiotic-specific (seed, β-lactam, and *E. coli* in ref. [40]) and cannot yet be generalized to leaves; for MRSA, only a single stem–bark/ampicillin study exists [41]. A reduced bacterial burden in turn lowers PAMP release and weakens upstream TLR4/NF-κB activation.

### 5.2. Anti-Inflammatory Action Restrains Progression and Protects Tissue

After bacteria and their toxins are brought under control, the constituents detailed in Section 4 restrain hyperinflammation and tissue injury through NF-κB inhibition, Nrf2 activation, pro-inflammatory enzyme blockade, and the control of macrophage polarization. Together, these effects (i) forestall progression to systemic inflammatory response syndrome or sepsis, (ii) curtail collateral damage from reactive oxygen species and proteases, and (iii) foster an M2/TGF-β/IL-10 milieu that favors repair [17]. In a rat model of infected diabetic ulcers, leaf-extract treatment lowered bacterial burden and wound TNF-α/IL-1β, raised IL-10, and accelerated closure, which are evidence of coordinated benefit even though formal synergy testing was not performed [32,71].

### 5.3. Cooperation Among Constituents and Across Pathways

At the constituent level, fatty acids, sterols, and cationic proteins perturb bacterial membranes, which should in principle facilitate the entry of flavonoids, phenolic acids, and ITCs; this sequence is inferred mainly from fraction analysis and ultrastructural observation, and direct time-resolved, stepwise-addition tests are still lacking [23,34]. In host immune cells, by contrast, flavonoid and ITC uptake depends mainly on passive diffusion and transporters and is not coupled to bacterial membrane permeabilization [4,17]. At the pathway level, antibacterial action reduces continued PAMP release and weakens TLR4/MyD88/NF-κB input, conversely, which would be expected to sensitize cells to NF-κB inhibition [69]; conversely, Nrf2-dependent antioxidant enzymes and intrinsic radical scavenging lower oxidative stress in the inflammatory microenvironment [16,23]. The hypothesis that antioxidant action protects oxidation-sensitive antibacterial compounds is biochemically reasonable but remains to be tested directly [27,57]. This two-way interaction offers a plausible basis for the broader activity of crude extracts relative to isolated compounds, rather than proof of superiority. Rapid bacteriolysis can briefly release large amounts of Gram-negative LPS (endotoxin release), and the mostly moderate, bacteriostatic character reported for these extracts may limit that effect. In Table 3, we summarize the cross-target matrix of major *M. oleifera* constituents, listing their antibacterial and anti-inflammatory targets, the highest tier of supporting evidence, and whether both bioactivities have been validated within a single experimental system. Notably, nearly all individual constituents have been assayed separately for antibacterial or anti-inflammatory effects, while simultaneous evaluation of both functions for a given compound remains scarce.

### 5.4. Temporal and Therapeutic Interplay and Evidence Gaps

Animal models of skin/soft-tissue and systemic infection suggest that the two activities carry different weight over the course of infection. Within the first 24 h, bacterial load rises rapidly and antibacterial mechanisms predominate. Inflammation peaks between roughly 24 and 72 h, when anti-inflammatory effects strengthen through NF-κB inhibition and Nrf2 activation. Beyond 72 h, bacterial load declines and tissue repair takes over, with macrophage infiltration, upregulation of vascular endothelial growth factor (VEGF) and TGF-β1, collagen deposition, and neovascularization [32,72]. This temporal division rests mainly on the rat infected diabetic-ulcer and mouse excisional-wound models and may differ with infection type, pathogen, and host status [32].

At the therapeutic level, a combined profile could lower the required antibiotic dose, cover a broad pathogen spectrum, address infection and inflammation at the same time, and in theory slow resistance through multitarget action; this last claim requires serial-passage resistance-selection experiments and clinical data [3,73]. Direct quantitative proof of antibacterial–anti-inflammatory synergy is still lacking. We found no study that quantified both activities and their interaction in a single system using a combination index (CI), isobologram, or response-surface analysis; synergy indices must be computed within one endpoint class—fractional inhibitory concentration indices from checkerboard and time–kill assays for bacterial growth, and a separately derived combination index for cytokine or enzyme readouts in cell assays—and must not be combined across bacterial and host endpoints; agents, fixed ratios, and dose ranges must be defined before analysis. Suitable designs are checkerboard assays with time–kill confirmation and isobolograms for antibacterial combinations, and factorial or response-surface designs (Chou–Talalay or Bliss independence) for anti-inflammatory combinations in a shared cell or animal system. Earlier statements implying a measured CI below 1 were not supported by the cited reports and have been removed. In vivo comparisons showing better survival, bacterial clearance, and histology with extracts than with a single antibiotic or a single anti-inflammatory drug provide suggestive, but not formal, evidence of interaction [32]. Future work should combine transcriptomics, proteomics, and metabolomics with mathematical modeling to quantify the topology and key nodes of any multicomponent network, as a basis for standardized preparations and rational use.

**Table 3:** Cross-target matrix linking principal *M. oleifera* constituents to their antibacterial and anti-inflammatory targets, the highest evidence tier reached, and whether both activities have been co-demonstrated in a single experimental system. Seed-derived constituents are labeled. “No” indicates that the two activities were reported in separate studies, so constituent-level cross-kingdom synergy remains untested. Evidence ranks from molecular docking (lowest) through in vitro enzyme/cell assays to animal and human studies (highest), as defined in Section 1.3.

Constituent (Principal Part)	Antibacterial Target/Action	Anti-Inflammatory Target/Action	Highest Evidence Tier	Both Activities Co-Tested in One System?
Flavonols: quercetin, kaempferol (leaves)	Membrane perturbation; predicted GyrB/ParE binding (docking); QS/agr inhibition	NF-κB p65/DNA binding; COX/LOX; Nrf2	In vitro + animal	No (separate studies)
Flavonoid glycosides: astragalin, rutin, hyperoside (leaves)	Membrane and biofilm effects; *E. coli* IC50 ≈ 0.21–0.36 mg/mL	Radical scavenging; weaker NF-κB effects	In vitro	No
Phenolic acids: chlorogenic, caffeic, gallic (leaves, stems)	Membrane; proposed cell-wall/ribosomal effects	NF-κB, iNOS/COX; radical scavenging	In vitro	No
GMG-ITC (moringin) (mainly seeds; seed-derived)	Biofilm/QS; membrane; energy-enzyme effects proposed	Keap1 cysteines/Nrf2; proposed IKKβ Cys179 (extrapolated from other ITCs); iNOS	In vitro + animal	No (separate studies)
Sterols: β-sitosterol, stigmasterol (leaves/stems/seeds)	Membrane insertion and leakage	Weak immunomodulatory effects	In vitro	No
Pentacyclic triterpenes: lupeol (leaves/stems)	Limited direct antibacterial data	NF-κB-directed effects reported mainly outside *M. oleifera*	Preliminary/analogy	No
Cationic protein/polypeptide MOCP (seeds; seed-derived)	Membrane fusion and leakage	Not established for leaves/stems	In vitro (seeds)	No
Native chitin-binding proteins: Mo-CBP2/3/4 (seeds; seed-derived)	Mainly fungal cell-wall chitin; limited antibacterial support	Not established	In vitro (antifungal)	No
Designed peptides: Mo-CBP3-PepI/II/III (synthetic; not native)	Membrane permeabilization; ROS induction	Not assessed	In vitro	No

## 6. Isolation, Identification, and Structure–Activity Relationships

### 6.1. Extraction and Isolation

Methods used to extract active constituents from leaves and stems include solvent reflux, ultrasound-assisted extraction (UAE), microwave-assisted extraction (MAE), and supercritical-CO2 extraction (SFE-CO2) (Figure 5). The methods differ in how efficiently they enrich each class. In several studies, UAE gave a higher total yield and higher total phenol and flavonoid content than conventional maceration or Soxhlet [74,75]. MAE is rapid but requires power control to avoid degrading heat-sensitive compounds [76]. Soxhlet extraction gave a lower total yield but enriched weakly polar flavonoid aglycones such as kaempferol [77]. Aqueous ethanol (50–80%) is efficient for recovery of flavonoids and phenolic acids. In contrast, n-hexane, petroleum ether, or supercritical-CO_2_ fluid extraction are preferred for sterols and fatty acids; notably, SFE-CO_2_ produces extracts free of organic-solvent residues [78,79].

Isolation typically begins with liquid–liquid extraction for preliminary fractionation, followed by column chromatography on silica gel, polyamide, or Sephadex LH-20 and high-speed counter-current chromatography (HSCCC) for fraction cutting, with preparative HPLC or supercritical-fluid chromatography for final monomer purification [80,81]. HSCCC offers preparative-scale, carrier-free separation of polyphenols, and its recoveries for *M. oleifera* flavonoids and phenolic acids should be cited from the primary separation study [80].

ITCs require specific handling. They are stored as glucosinolate precursors and released only when tissue disruption brings them into contact with myrosinase. Two extraction strategies are commonly employed, depending on whether the target is intact glucosinolates or their ITC products [82,83]. The first hydrolyzes glucosinolates before extraction: after tissue disruption, the material is incubated at 25–40 °C and an appropriate pH to allow endogenous myrosinase to convert glucosinolates into ITCs, followed by low-temperature extraction with water or dilute alcohol. The second inactivates myrosinase first, using boiling water or high-concentration methanol, to preserve glucosinolate precursors, with exogenous myrosinase added only immediately before use. Because they carry a rhamnose moiety, *M. oleifera* ITCs such as moringin are more stable than lipid-soluble ITCs such as sulforaphane, but temperatures above 40 °C, light, and prolonged exposure to oxygen still cause degradation; extraction and concentration should therefore use low temperatures (≤40 °C), light protection, and short processing times [20,82,84].

### 6.2. Structure–Activity Relationships

#### 6.2.1. Flavonoids

Free phenolic hydroxyls, especially the B-ring ortho-dihydroxyl (3′,4′-catechol) group, are central to antioxidant and antibacterial activity. The conjugated C2=C3 bond and the 4-carbonyl maintain planarity and delocalize electrons, strengthening target binding [15,85]. Glycosylation at C3 alters solubility, stability, and oral bioavailability. Glucosides such as isoquercitrin can be hydrolyzed to the aglycone by small-intestinal lactase-phlorizin hydrolase (LPH) and then absorbed, whereas rhamnosides such as quercitrin are not recognized by LPH and require colonic β-rhamnosidase, leading to slower absorption and lower bioavailability [86,87,88]. Glycosylation usually lowers direct antibacterial activity by masking free phenolic hydroxyls and increasing polarity, while raising water solubility and in vivo stability, so the net effect depends on the target and route [85]. Greater planarity favors passive diffusion across both bacterial and host membranes.

#### 6.2.2. Phenolic Acids

The number of phenolic hydroxyls generally tracks antioxidant and antibacterial activity. With three adjacent hydroxyls, gallic acid (3,4,5-trihydroxybenzoic acid) was more active than mono- or di-hydroxyl acids such as p-coumaric and caffeic acids in several assays [89,90]. Esterification or glycosylation of the carboxyl group increases lipid solubility and membrane penetration but may weaken hydrogen-bond and ionic interactions, so the effect is target-dependent: esterified derivatives were often favored in membrane-targeted antibacterial assays, whereas a free carboxyl may matter more in enzyme inhibition [4,91,92].

#### 6.2.3. Isothiocyanates

The –N=C=S group is the pharmacophore: its electrophilic central carbon reacts with the thiols of protein cysteines and the amines of lysines to regulate NF-κB and Nrf2 and to contribute antibacterial action [36,93,94]. The para α-L-rhamnosyloxy group of moringin confers water solubility and stability. Acetylation of sugar hydroxyls, as in 4′-O-acetylmoringin, increases lipid solubility and membrane penetration, and some reports describe greater uptake or activity, although head-to-head comparisons remain limited [82,95].

#### 6.2.4. Sterols

Plant sterols such as β-sitosterol and stigmasterol show weak overall antibacterial activity in reported assays, linked mainly to membrane insertion and altered fluidity [96,97]. The proposed rule that more double bonds and a longer C17 side chain strengthen antibacterial activity appears only as a trend across a few comparisons, lacks systematic quantitative structure–activity relationship (QSAR) support, and should not be treated as settled.

Overall, structure–activity work on *M. oleifera* constituents remains limited: most conclusions rest on simple comparisons of related compounds or on docking, rather than on systematic QSAR and site-directed modification, and cooperation among constituents in crude extracts means that monomer structure–activity relations cannot fully account for whole-extract activity. Combinatorial-chemistry and metabolomics approaches are therefore needed [85].

## 7. In Vivo Metabolism and Safety Evaluation

### 7.1. Pharmacokinetic Characteristics

Oral absorption varies by class. ITCs, sterols, and flavonoid aglycones are more lipid-soluble and have relatively better bioavailability, whereas flavonoid glycosides and phenolic acids are absorbed less efficiently [14,98]. Reported distribution differs between parent compounds and their glucuronide/sulfate conjugates and between in silico prediction and experimental pharmacokinetics, which must not be conflated; experimental data place absorbed flavonoid conjugates mainly in the circulation, liver, and kidney [99,100].

Whether ITCs slow flavonoid metabolism and prolong their residence in mixtures requires direct pharmacokinetic evidence [16].

### 7.2. Toxicological Evaluation and Safe Doses

Safety has been examined in several studies, but a clear distinction must be drawn between acute single-dose tests and repeated-dose or chronic safety. In acute oral toxicity tests (single or 24 h dosing; for example, OECD Test Guideline 423), aqueous and ethanol leaf extracts were generally classified as low-toxicity, with LD50 values above 2000 mg/kg in rodents (values vary with solvent and species) [101,102,103,104]. A repeated-dose 13-week study of an optimized aqueous leaf extract in mice provided subchronic information [105], and a purified seed lectin (cMoL) was assessed for acute and genotoxic toxicity (seed-derived) [106]. Subacute repeated-dose studies (14–28 days) reported no overt organ toxicity at lower doses, with mild liver-enzyme (ALT/ALP) elevation at high doses (at 800–1600 mg/kg) [104]; a 90-day subchronic study at up to 600 mg/kg reported no hepatic-enzyme changes [107].

### 7.3. Herb–Drug Interactions

ITCs and flavonoids can inhibit drug-metabolizing enzymes such as CYP3A4, CYP2D6, and CYP2C19 in vitro. When combined with drugs metabolized by these enzymes (calcium-channel blockers, antidepressants, and proton-pump inhibitors), they could in theory slow metabolism and raise plasma concentrations, but clinical interaction data are lacking [14,98,108,109]. Their reported hypoglycemic and hypotensive activities may add to those of related drugs; monitoring and avoidance of high-dose, long-term combination use are prudent while interaction studies remain pending [66,98].

## 8. Current Status and Future Perspectives

On the basis of the published worldwide work, several conclusions can be drawn (Figure 6). First, the antibacterial and anti-inflammatory activities of leaves and stems have reasonable scientific support: extracts, fractions, and monomers show broad-spectrum inhibition of Gram-positive and Gram-negative bacteria and some fungi, and the antibacterial–anti-inflammatory interplay is a plausible advantage, although formal synergy remains to be demonstrated [9,14]. Second, more than 160 compounds have been reported across the whole plant, with a smaller documented bioactive subset; the structure–activity features and multitarget mechanisms of the five core classes (flavonoids, phenolic acids, ITCs, sterols, and peptides) are partly defined, whereas systematic QSAR work remains scarce [4,17,110]. Third, leaves and stems show application potential in medicine, animal production, food, and cosmetics, with pilot-scale exploration in feed and food but an early overall stage of translation [111,112,113].

### 8.1. Application Areas and Translation Prospects

1. Clinical treatment. Extracts are active against MDR bacteria and show a trend toward additive effects with some antibiotics, with part- and antibiotic-specific evidence (Section 3.3). In a diabetic-rat excisional-wound model, topical leaf methanol extract promoted healing of MRSA/*P. aeruginosa*-infected wounds through antibacterial and antioxidant action and VEGF/TGF-β1 upregulation [32,114]. The anti-inflammatory activity, mediated by dual NF-κB/Nrf2 regulation, is relevant to infection-related inflammation as adjunctive therapy, but the human evidence remains small-sample and exploratory (Section 4.5).
2. Animal production. Leaf meal is being explored as a natural feed additive in place of antimicrobial growth promoters. Poultry trials, including an integrated microbiome and metabolome study in meat ducks, report improved growth and carcass traits and favorable shifts in the gut microbiota (lower enterobacteria, higher *Lactobacillus*/*Bifidobacterium*), although the microbiota endpoints and their magnitude vary among studies [115,116,117,118]. In sows and piglets, dietary leaf meal improved antioxidant status and performance during late gestation and lactation and after weaning [119,120]. Because of crude fiber and antinutritional factors, inclusion levels for monogastric animals are generally kept low (often around or below 10%) [113].
3. Food preservation. Leaf and stem extracts inhibit *E. coli* and *S. aureus* and slow lipid oxidation in foods during cold storage [75,121]. Flavonoids and phenolics inhibit lipid oxidation and have been compared to synthetic antioxidants such as butylated hydroxytoluene (BHT) [122]. They offer a natural-source advantage, although chlorophyll and phenolics can alter color and flavor.
4. Cosmetics and oral care. Extracts can serve as natural preservatives and functional ingredients, with in vitro inhibition of collagenase, elastase, and hyaluronidase and reported topical improvement in erythema and hydration [4,123,124,125]. In oral care, a randomized crossover trial found that *M. oleifera* dentifrice reduced plaque and gingivitis relative to the control dentifrice in that trial [126]. Acne and eczema remain plausible but clinically under-verified targets.

### 8.2. Research Limitations

The principal limitations, beyond the scope and evidence-grading issues noted in Section 1.3 and Section 5, are as follows. (1) Most work remains at the crude-extract level: monomer isolation, structure–activity relations, and multicomponent synergy or antagonism are understudied, and synergistic ratios and dose–response relations are undefined [4,16,127]. (2) High-quality human evidence is lacking: studies are small, with no multicenter randomized double-blind trials and no unified dosing or efficacy standards; an umbrella review of 26 systematic reviews rated the available evidence quality as mostly low to very low [66,128]. (3) PK/PD and toxicological coverage is incomplete, especially across populations and disease states, and reproductive and genotoxic data are limited [14,129]. (4) Preparation standardization is low: region, harvest, and processing alter content, no unified international pharmacopeial standard exists, and standardized active-fraction or monomer preparations are lacking [1,110]. (5) Antibacterial–anti-inflammatory synergy is described mostly qualitatively, without CI, isobologram, or response-surface quantification, defined ratios, or temporal dose–response relations. (6) Targeted in vivo distribution and metabolic tracking at infection foci (lung, skin ulcer, and intestine) are limited, making in vitro–in vivo translation difficult. (7) The gut microbiota–host axis is under-examined: polyphenol and ITC activity in vivo may depend on microbial transformation (glucosinolate hydrolysis and glycoside deglycosylation) and on microbiota/short-chain fatty acid changes that have not been studied systematically in *M. oleifera* [130]. (8) Much of the strongest mechanistic evidence for ITCs and cationic peptides originates from seeds, whereas leaves and stems differ in constituent profile and content, so seed-derived efficacy cannot be assumed for leaf or stem preparations without direct measurement. (9) Antibacterial and anti-inflammatory studies use different organisms, readouts, and units (bacterial growth versus host cytokines), and no unified cross-kingdom platform yet measures both in one system; together with the lack of standardized multi-constituent reference materials, this impedes direct comparison and synergy assessment.

### 8.3. Future Directions

We suggest the following priorities in descending order: near-term standardization of leaf/stem preparations and direct synergy testing (items 1, 4, and 5); mid-term PK/PD, targeted delivery, and microbiota work (items 3, 6, and 7); and longer-term high-quality clinical and antibiotic-combination studies (items 2 and 8). (1) Chemistry and preparations. Systematically isolate monomers, define multicomponent interactions, and establish activity-guided extraction and multi-marker quality standards. (2) High-quality clinical research. Multicenter randomized double-blind trials are needed to establish efficacy, safe doses, and adverse effects under unified regimens and endpoints [66]. (3) Systematic PK/PD and toxicology. Quantify the absorption, distribution, metabolism, and excretion of principal constituents, develop PK/PD models, and conduct long-term, reproductive, and genotoxic toxicity studies to underpin clinical safety assessment [14,98]. (4) Indication-driven mechanism work. With resistant infection and chronic inflammation as targets, test constituents alone and with antibiotics and resolve combined-action signaling [9]. (5) Multi-omics of interplay. Use transcriptomics, proteomics, metabolomics, and microbiomics to map the infection–inflammation network and identify synergy biomarkers for multicomponent quality control [131]. (6) Targeted delivery. Develop nanoliposomes, nanoemulsions, mesoporous silica, and cyclodextrin carriers (pH-/ROS-responsive and macrophage-targeted) to raise local concentration and lower systemic exposure [132,133]. (7) Microbiota-mediated effects. Combine in vitro gut fermentation, animal models, and human verification of how the microbiota activate precursors such as GMG; an in vivo seed-ITC study showed microbiota modulation (seed-derived) [134], but the specific in vitro conversion of GMG to moringin by *Lactobacillus*/*Bifidobacterium* still requires a dedicated microbial-biotransformation study. (8) Antibiotic-combination translation. For priority resistant pathogens (MRSA, ESBL-producing Enterobacterales, and carbapenem-resistant strains), run in vivo infection, and phase I/II studies [41]; synergistic ratios, dose ranges, and safety margins remain to be defined.

## Figures and Tables

**Figure 1:**
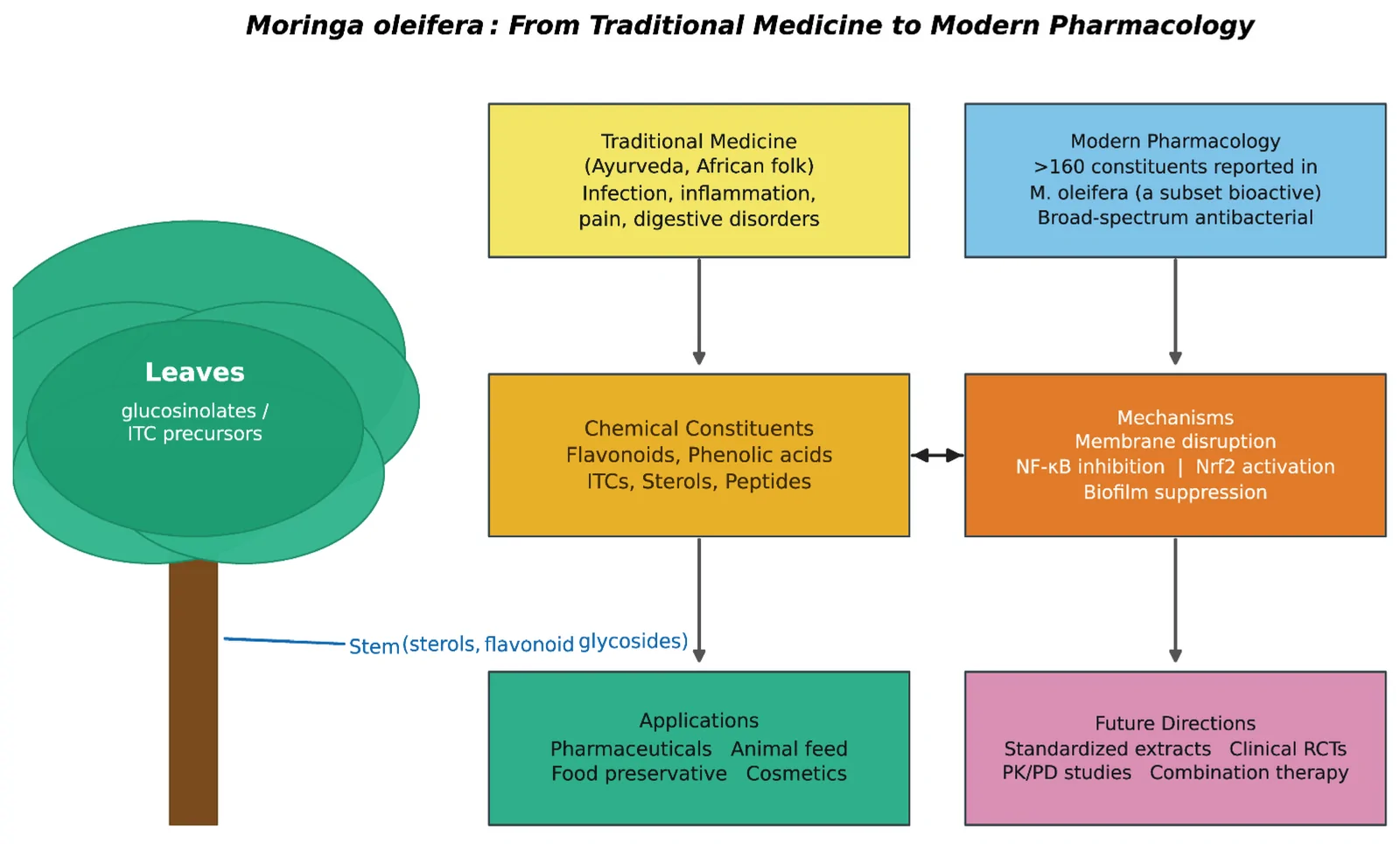
Research overview of *Moringa oleifera* Lam., from traditional use to modern pharmacology. The scheme links the traditional applications of leaves and stems to their principal constituent classes (flavonoids, phenolic acids, isothiocyanates, sterols, and peptides), to the reported antibacterial and anti-inflammatory activities and their molecular targets, and to the application areas (medicine, animal production, food preservation, and cosmetics) discussed in this Review.

**Figure 2:**
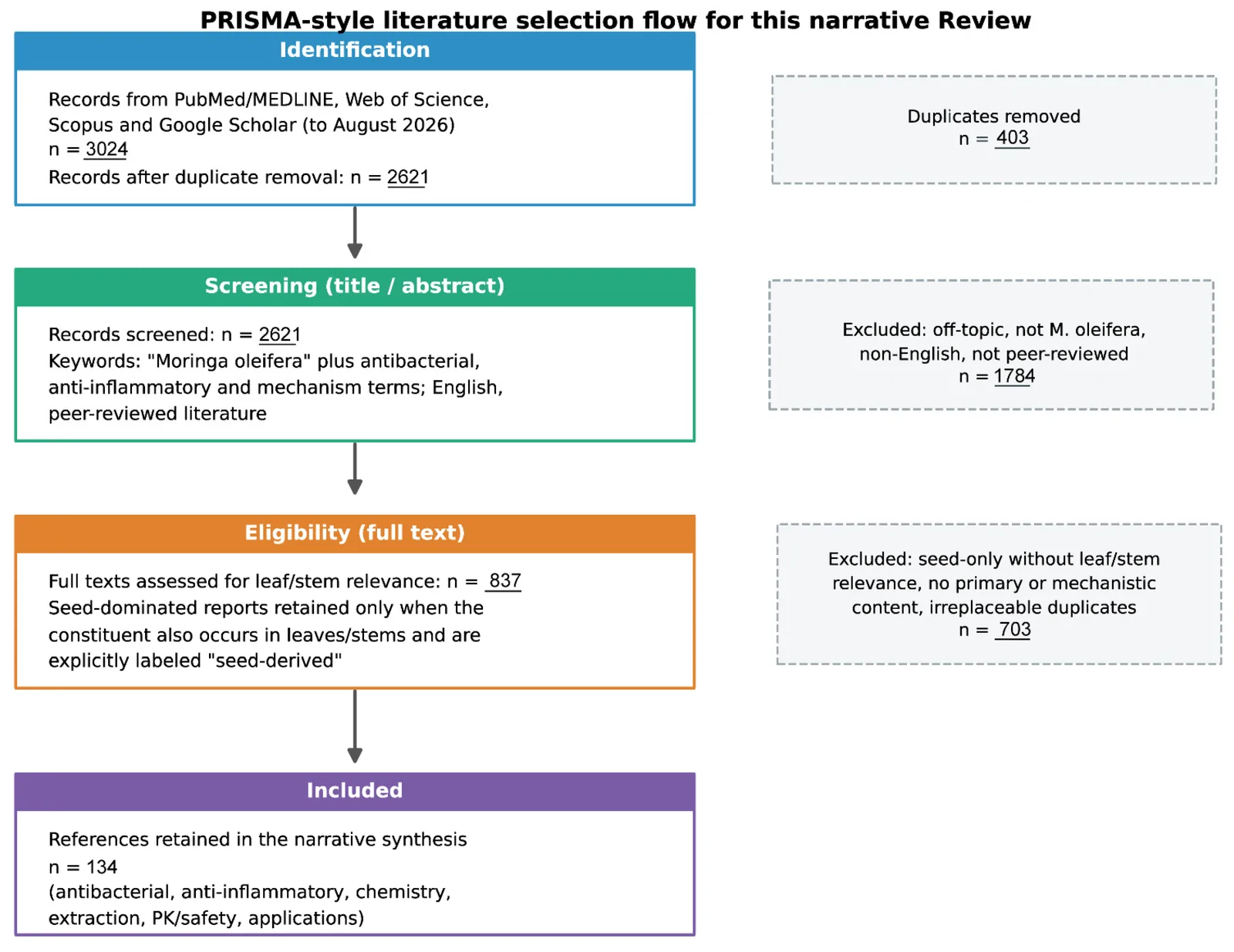
PRISMA-style literature selection flow for this narrative Review. Records were retrieved from PubMed/MEDLINE, Web of Science, Scopus, and Google Scholar up to August 2026; after duplicate removal and title/abstract screening for relevance to *Moringa oleifera* leaves and stems, 134 references were retained. In the flow diagram, the underlined values are the record counts retained at each screening stage.

**Figure 3:**
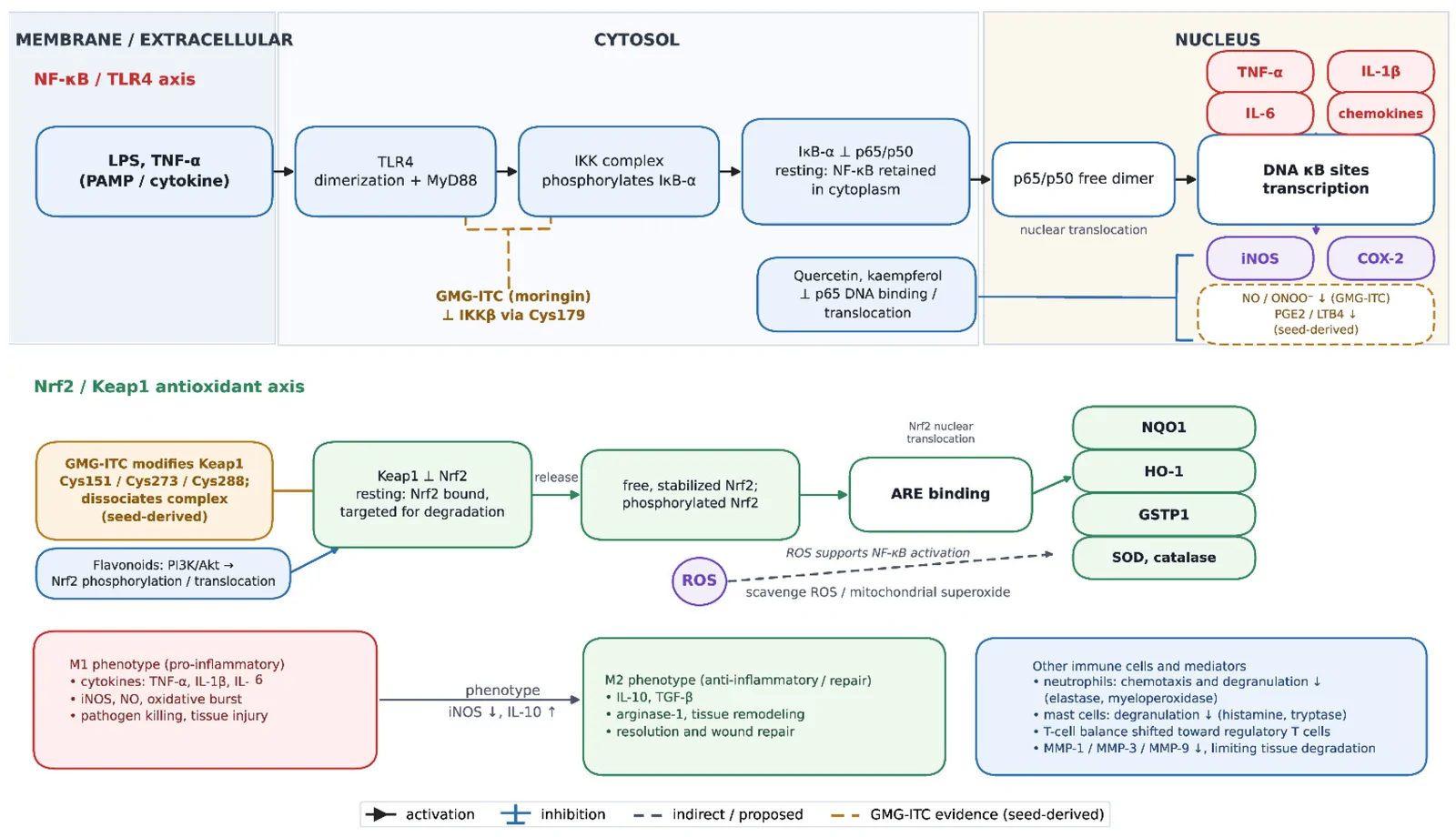
Anti-inflammatory mechanisms of *M. oleifera* leaf and stem constituents in macrophage NF-κB and Nrf2/Keap1 signaling. The diagram shows inhibition of the NF-κB/TLR4 pro-inflammatory pathway, activation of the Nrf2/Keap1 antioxidant axis, inhibition of pro-inflammatory enzymes and mediators (COX/LOX, iNOS, and MMPs), and modulation of macrophage polarization and other immune cells. Arrow key: ↑ indicates activation/up-regulation, ↓ indicates inhibition/down-regulation.

**Figure 4:**
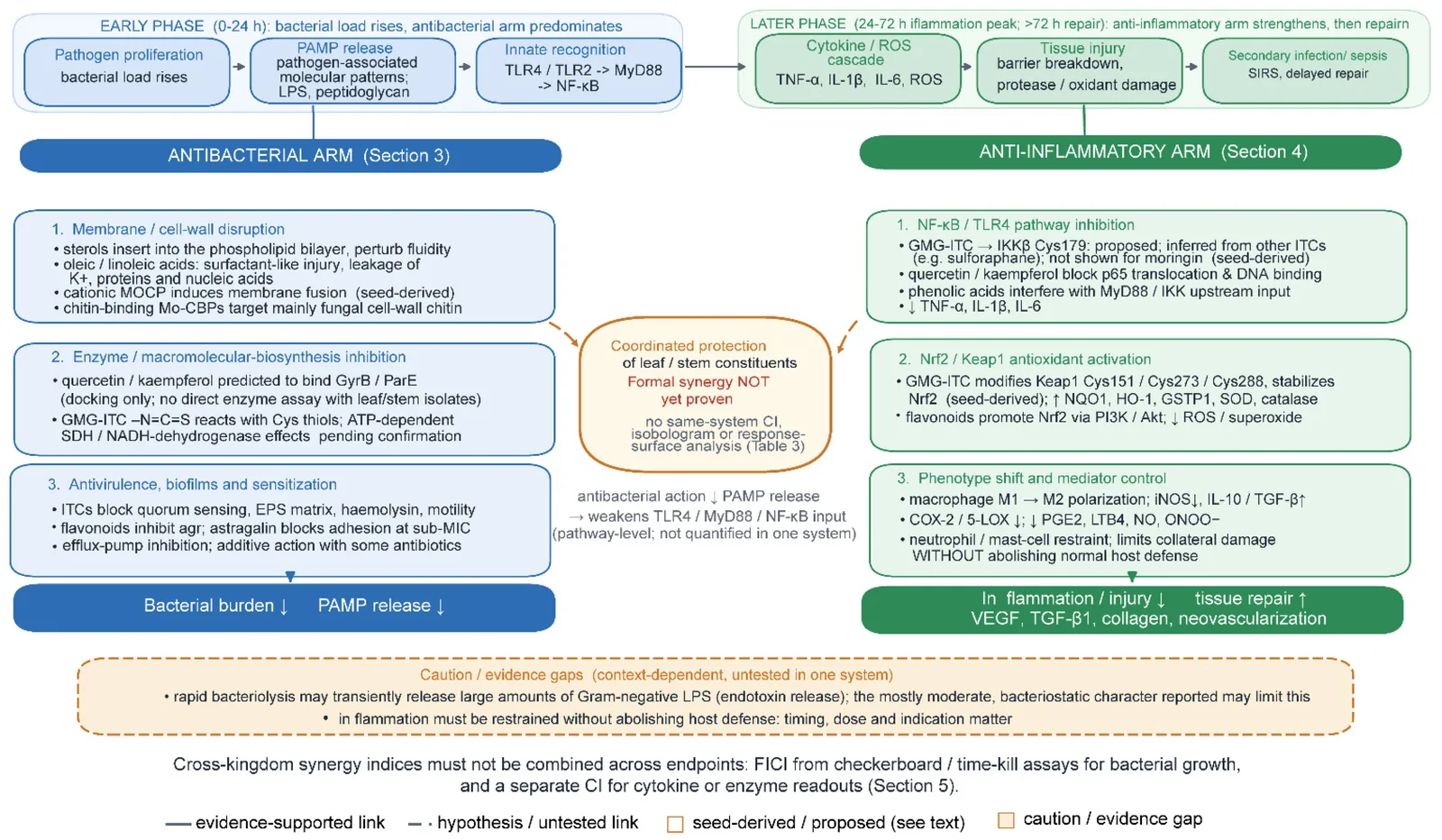
Proposed interplay between the antibacterial and anti-inflammatory actions of *M. oleifera* leaf and stem constituents. The scheme depicts antibacterial action reducing pathogen-associated molecular pattern (PAMP) release, anti-inflammatory action restraining NF-κB-driven tissue injury, constituent- and pathway-level cooperation, and the proposed temporal sequence over the course of infection; demonstrated interactions are distinguished from mechanistic hypotheses. Arrow key: ↑ indicates activation/up-regulation, ↓ indicates inhibition/down-regulation.

**Figure 5:**
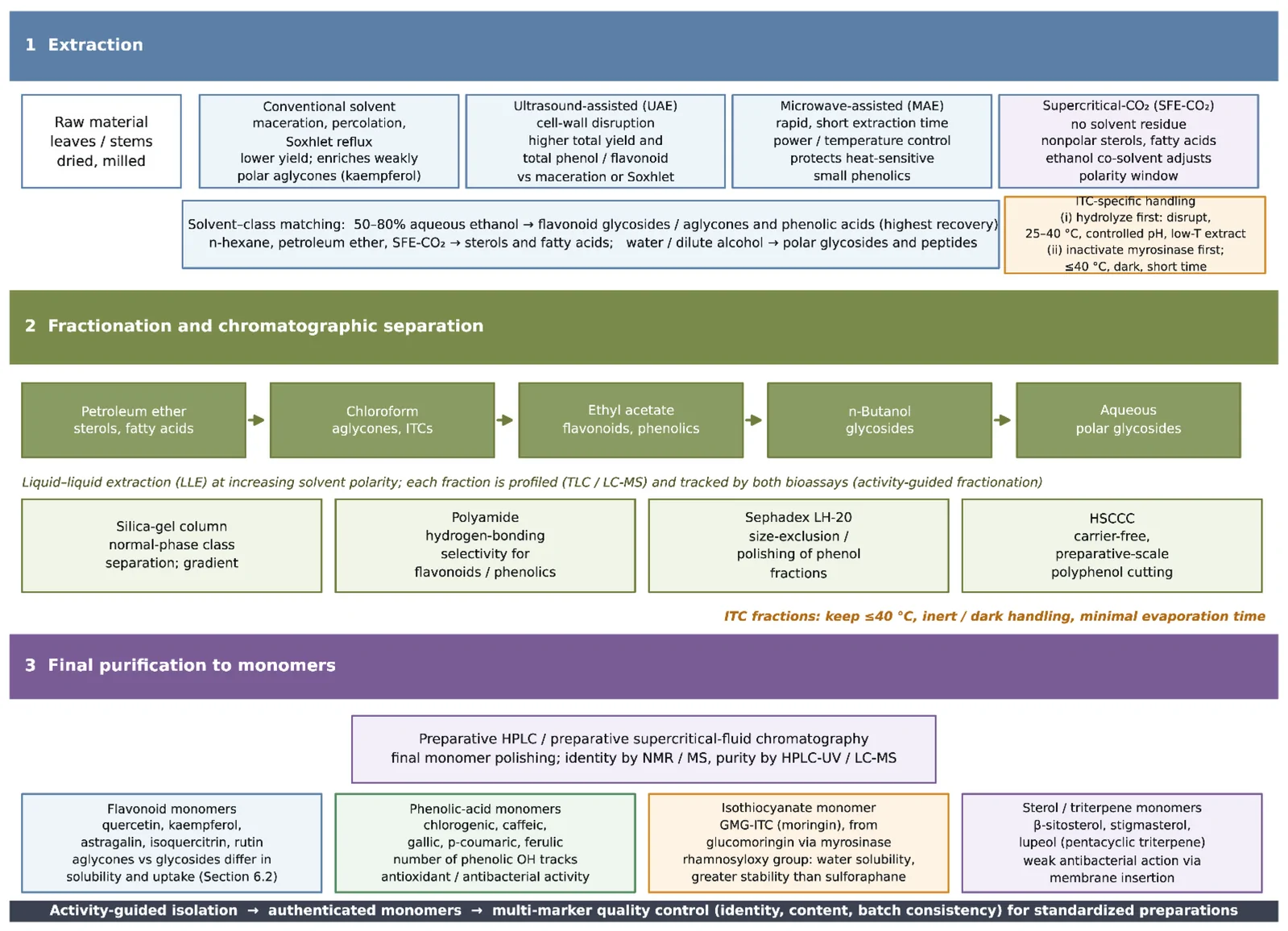
Systematic isolation and purification strategy for *M. oleifera* active constituents, from raw material to monomers. The workflow covers solvent selection and extraction (reflux, ultrasound-assisted, microwave-assisted, and supercritical-CO2 extraction), liquid–liquid fractionation, column and counter-current chromatographic separation, and final monomer purification, together with the specific handling required for glucosinolate/isothiocyanate interconversion.

**Figure 6:**
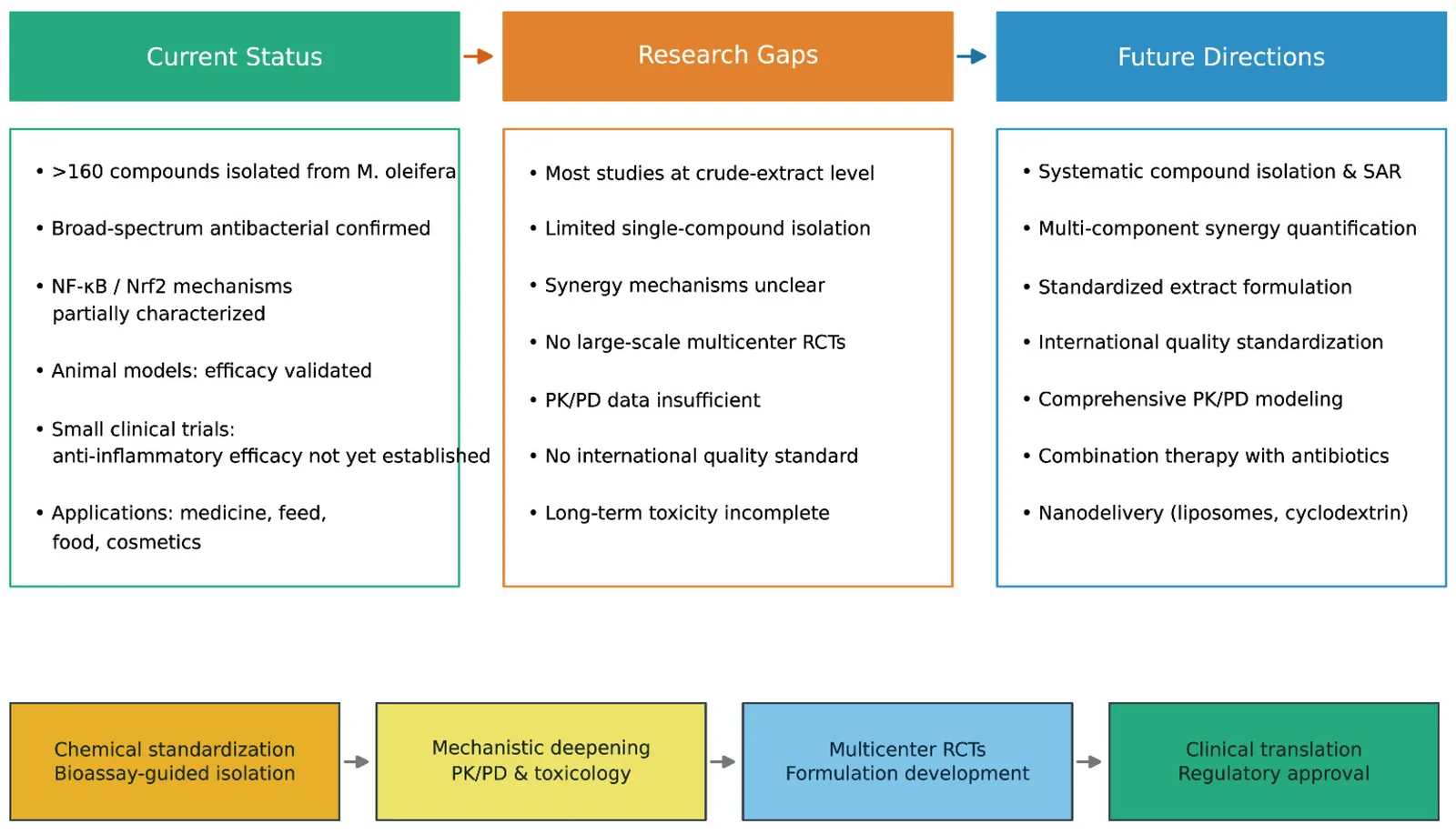
Current status, gaps, and future directions for research on the antibacterial and anti-inflammatory activities of *M. oleifera* leaves and stems. The scheme summarizes current status, the principal limitations (preparation standardization, synergy testing, PK/PD and safety, and clinical evidence), and the prioritized research roadmap.

**Table 1:** Classification and structural features of the principal active constituents of *M. oleifera* leaves and stems.

Compound Class	Representative Constituents	Core Structural Features	Main Activity	Principal Plant Part(s)
Flavonoids	Quercetin, kaempferol, astragalin, isoquercitrin, rutin, hyperoside	Flavonol skeleton; phenolic hydroxyls at C3/C5/C7; mainly O-glycosides	Antibacterial, anti-inflammatory, antioxidant	Leaves (dominant), stems (glycosides)
Phenolic acids	Chlorogenic, caffeic, gallic, p-coumaric, ferulic acids	Benzoic- or cinnamic-acid skeleton; carboxyl with multiple phenolic hydroxyls	Antibacterial, anti-inflammatory, antioxidant	Leaves, stems
Isothiocyanates (ITCs)	GMG-ITC (moringin)	–N=C=S group; benzyl-linked rhamnoside; released from glucomoringin by myrosinase	Anti-inflammatory, antibacterial, antioxidant	Best characterized in seeds; also present in leaves
Sterols	β-Sitosterol, stigmasterol	Cyclopentanoperhydrophenanthrene skeleton; C3-OH	Weak antibacterial, anti-inflammatory, immunomodulatory	Leaves, stems, seeds
Pentacyclic triterpenes	Lupeol	Pentacyclic C30 triterpene skeleton; distinct from the tetracyclic cyclopentanoperhydrophenanthrene sterol nucleus	Weak/indirect anti-inflammatory and antibacterial support; previously grouped with sterols	Leaves, stems
Chitin-binding proteins (native)	Mo-CBP2, Mo-CBP3, Mo-CBP4	Chitin-binding domain; cationic, cysteine-rich proteins	Mainly antifungal; only limited direct antibacterial support	Mainly seeds; leaf/stem abundance not yet characterized (seed-derived)
Designed antimicrobial peptides (synthetic)	Mo-CBP3-PepI, Mo-CBP3-PepII, Mo-CBP3-PepIII	Short cationic peptides designed from the Mo-CBP3 sequence	Antibacterial; plasma-membrane permeabilization and ROS induction	Synthetic constructs; not native leaf or stem constituents

Note. “Principal plant part(s)” indicates where each class has been characterized most thoroughly, rather than its exclusive site of occurrence. Hyperoside is listed among the flavonoids because it was one of four *M. oleifera* polyphenol monomers tested against *E. coli* in ref. [15].

**Table 2:** Reported antibacterial activity of *M. oleifera* leaf and stem extracts against major pathogenic bacteria.

Material (Plant Part; Extraction)	Strain (Gram Class)	MIC (mg/mL)	Inhibition-Zone Diameter (mm)	Ref(s)
Leaf; ethanol/methanol	*E. coli* (G−)	0.25–4.0 (pooled range)	n.r.	[16,21,23]
Leaf; ethanol/methanol	*S. aureus* (G+)	0.25–4.0 (pooled range)	n.r.	[21,22]
Leaf; methanol	*K. pneumoniae* (G−)	2.0–6.0 (pooled range)	n.r.	[16,21]
Leaf and seed (seed-derived)	*MRSA* (G+)	n.r. (qualitative)	Qualitative inhibition	[27,32]
Leaf; aqueous	*B. cereus* (G+)	0.33	7.7 (disk diffusion)	[21]
Leaf; aqueous/alcohol	*E. faecalis* (G+)	n.r. (qualitative)	Qualitative inhibition	[21,33]
Astragalin (pure monomer)	*E. coli* (G−)	IC50 ≈ 0.22 (490 μmol/L)	n.r.	[15]
Rutin (pure monomer)	*E. coli* (G−)	IC50 ≈ 0.36 (588 μmol/L)	n.r.	[15]
Chlorogenic acid (pure monomer)	*E. coli* (G−)	IC50 ≈ 0.21 (605 μmol/L)	n.r.	[15]
Hyperoside (pure monomer)	*E. coli* (G−)	IC50 ≈ 0.31 (676 μmol/L)	n.r.	[15]

Note. The MIC ranges pool studies that differ in solvent, geographic origin, inoculum, and assay (broth or agar dilution versus disk diffusion) and must not be read as a single distribution. “Qualitative” indicates that the source reported inhibition without a standardized MIC. “n.r.” indicates that a standardized value was not reported in the cited source.

## Data Availability

No new data were created or analyzed in this study. Data sharing is not applicable to this article.
